# Performance of ENMC and EULAR/ACR classification systems applied to a single tertiary center cohort of dermatomyositis patients

**DOI:** 10.1186/s42466-021-00159-4

**Published:** 2021-11-15

**Authors:** Jan Zoske, Udo Schneider, Elise Siegert, Felix Kleefeld, Corinna Preuße, Werner Stenzel, Katrin Hahn

**Affiliations:** 1 Department of Periodontology, Oral Medicine and Oral Surgery, Charité - Universitätsmedizin Berlin, Corporate Member of Freie Universität Berlin, Humboldt-Universität Zu Berlin, and Berlin Institute of Health, 14197 Berlin, Germany; 2 Department of Rheumatology and Clinical Immunology, Charité – Universitätsmedizin Berlin, Charitéplatz 1, 10117 Berlin, Germany; 3 Berlin Institute of Health (BIH), Anna-Louisa-Karsch-Str. 2, 10178 Berlin, Germany; 4 Department of Neurology, Charité - Universitätsmedizin Berlin, Charitéplatz 1, 10117 Berlin, Germany; 5 Department of Neuropathology, Charité - Universitätsmedizin, Corporate Member of Freie Universität Berlin, Humboldt-Universität Zu Berlin, and Berlin Institute of Health (BIH), Berlin, Germany

**Keywords:** Dermatomyositis, ENMC, EULAR/ACR, Idiopathic inflammatory myositis, Muscle biopsy

## Abstract

**Background:**

There have been numerous classification systems to diagnose corresponding myositis subtypes and select appropriate therapeutic measures. However, the lack of a broad consensus on diagnostic criteria has led to clinical uncertainties. The objective of this study was to compare two commonly used dermatomyositis-classification systems regarding their clinical practicability and to point out their specific advantages and disadvantages.

**Methods:**

This study included 30 patients diagnosed with dermatomyositis at the Charité university hospital, Berlin, Germany from 2010 to 2017. Patient files with complete data and defined historical classifications were enrolled and ENMC (2003) and EULAR/ACR (2017) criteria retrospectively applied.

**Results:**

According to the ENMC approach, 14 patients were classified as "definite" and 12 as "probable" dermatomyositis. One patient exhibited an "amyopathic dermatomyositis" and three a "DM without dermatitis". Regarding the criteria probability of the EULAR/ACR set, 16 patients had a "high", 13 a "medium" and one a "low probability". There was a significant difference (p = 0.004) between the subclasses of the ENMC in relation to the EULAR/ACR score. The agreement between the classification probabilities of "definite/high" (κ = 0.400) and "possible/medium" (κ = 0.324) was fair.

**Conclusions:**

It is important to find a consensus among the medical disciplines involved and to establish a structured procedure. Future studies with newer approaches are warranted to conclusively decide which system to use for the physician.

## Introduction

Idiopathic inflammatory myopathies (IIM) are rare heterogeneous diseases characterized by inflammation in the skeletal muscles and resulting muscle weakness [1]. They frequently involve other organs such as the skin, joints, lungs or heart [2–4]. Depending on the clinical phenotype, the histomorphological findings and the involvement of other organs, different subtypes can be classified. A widely accepted basis is the 1975 description by Bohan and Peter for polymyositis (PM) and dermatomyositis (DM) [5, 6]. They defined for the first time the need of a clear classification and created the basis for the development of modern myositis classification systems. Even today, the core statements of their criteria are still part of various classification systems and are still commonly used [7]. In 2003, the European Neuromuscular Centre (ENMC) international workshop (2003 ENMC–IIM) revised dermatomyositis, polymyositis and inclusion body myositis (IBM) classification and proposed two additional categories. These are called immune-mediated necrotising myopathy (IMNM) and non-specific myositis. The aim was to be able to further differentiate IIM according to a possible different pathogenesis [8, 9]. In 2017, new dermatomyositis classification criteria according to the European League against Rheumatism (EULAR) and the American College of Rheumatology (ACR) were proposed which were validated through 976 patients with idiopathic inflammatory myopathies as well as 624 non-myopathic controls [10, 11]. Historically, the classifications are subject to a dynamic process, which is mainly based on new findings in the fields of immunology and pathology [12, 13]. Over the last few decades, there have been numerous classification systems with different focuses. However, the lack of a broad clinical consensus has resulted in diagnostic uncertainties in clinical practice for many physicians, especially those who do not deal with the latest findings on a daily basis and need clear guidelines [12, 14]. This individual change in diagnostics is evident in the systems used and compared in this study. They are characteristic of the classification criteria that have been adapted in different ways.

Dermatomyositis can be clinically identified by a characteristic dermatological phenotype such as the heliotrope rash and Gottron's papules or Gottron's sign. The patients frequently exhibit a progressive symmetrical muscle weakness of the upper and lower proximal musculature. Nevertheless, this typical clinical picture can be considered rare with a fluctuating incidence of 2–9/1.000.000 depending on the population [15, 16]. Over the last few decades, several DM-specific autoantibodies have been discovered and each of these has been associated with a unique clinical phenotype [17, 18]. The different subtypes differ in terms of both therapeutic response and prognosis of the disease, so that a clear assignment is of considerable clinical relevance [19].

The aim of this study is to compare ENMC and EULAR/ACR classifications retrospectively.

## Materials and methods

### Study design

This was a retrospective study conducted in the departments of Neurology and Rheumatology of the Charité university hospital. All study procedures were in accordance with the 1964 Helsinki declaration and its later amendments. The Medical Ethics Committee had approved the study (EA4/053/17).

### Study population

**Fig. 1:**
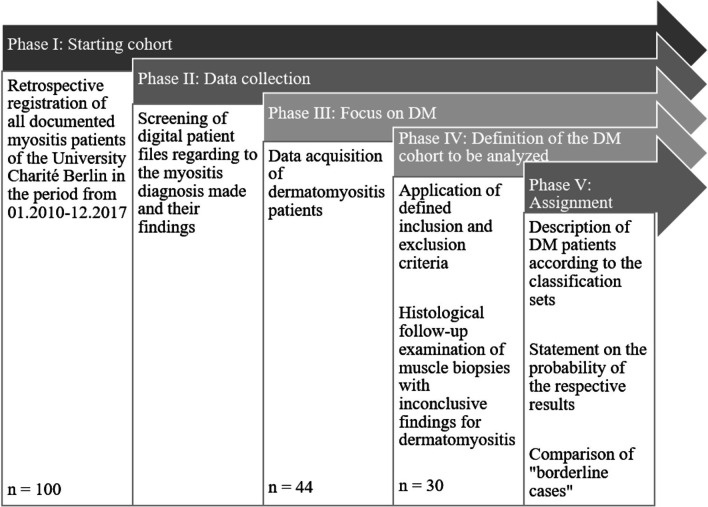
Graphical workflow of this study

We screened electronical hospital files from 2010 to 2017 retrospectively for “International Statistical Classification of Diseases and Related Health Problems” (ICD)-10 codes (M33.0, M33.1, M33.2, M33.9, M35.1, M60.8) corresponding to the different subtypes of myositis. Figure 1 summarizes the screening process.

To provide a solid basis for the selection of the patient collective, all myositis patient cases (n = 100) were reviewed with regard to the myositis diagnosis made and their findings. We identified 44 patients with suspected DM. Applicability of the ENMC and EULAR/ACR criteria was mandatory for our analysis. Patients with inaccurate, very incomplete or missing documentation were excluded. This also included patients whose DM diagnosis was changed during treatment. Patients who did not return after their first visit were also removed from our analysis. In the end, we obtained a strictly selected group of 30 DM patients.

### Outcome assessments

The same person carried out the data collection as well as the application of the classification systems.

**Fig. 2:**
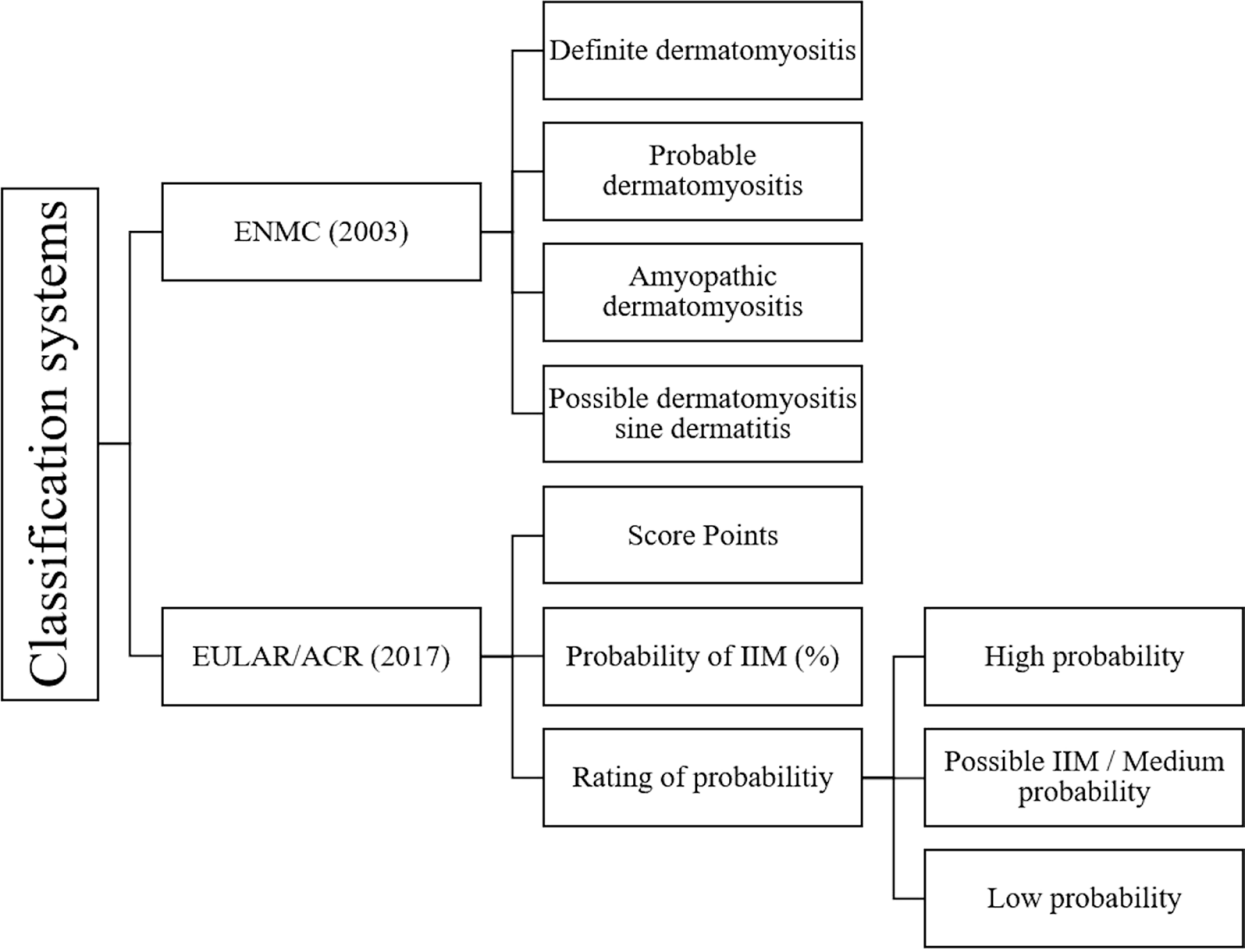
Overview chart to show the classification possibilities according to the ENMC (2003) and EULAR/ACR (2017) approaches and thus the classification clusters used in this study

The patient classification and, if included in the sets, the statement on the criteria probability were carried out strictly according to the specifications of the respective systems. Figure 2 summarizes the classification options.

### Patient classification according to ENMC (2003)

The decision on classification was based on the combination of clinical myogenic as well as clinical dermatological, histopathological, serological and other findings, like electromyography (EMG) or magnetic resonance imaging (MRI) [9]. A "definite dermatomyositis" can be classified if all clinical criteria are met and if perifascicular atrophy is found in the histopathological diagnosis. A "probable DM" is diagnosed by the presence of all clinical criteria and the presence of one of the three following findings: This includes the histopathological picture of a myopathic pattern and/or perivascular and perimysial inflammatory cell infiltrates. The second finding relates to elevated serum creatine kinase levels and the third to abnormal findings of the "other laboratory criteria" (EMG, electrocardiography, myositis-specific antibodies). "Amyopathic dermatomyositis" is classified according to the ENMC set if the following typical combination of clinical and laboratory findings can be described: Although a typical dermatological picture occurs, there is no objective muscle weakness. A normal serum CK and normal EMG findings can be detected. The muscle biopsy finding shows no abnormalities of a "definite" or "probable" DM. A characteristic feature is the finding of a reduced capillary density, the accumulation of membrane attack complex (MAC) on small blood vessels along the dermal–epidermal junction and a variable keratinocyte decoration in the examination of a skin biopsy. The last subclass of "possible DM *sine dermatitis*" is classified if a typical pattern of histopathological (perifascicular atrophy; MAC deposits), serological and "other laboratory criteria" and clinical findings (excluding dermatological characteristics) is observed.

### Patient classification according to EULAR/ACR (2017)

The use of the EULAR/ACR set was carried out in the sense of the model system "with" or "without" the use of a muscle biopsy with 16 included variables [20]. Due to the pre-selection of the patient cohort, it was not necessary to use the EULAR/ACR classification tree to assign a subgroup of IIM. The Web-based calculator was used to assign findings to a score range, probability, and classification [21]. The most important aspects, namely the age of manifestation, the distribution pattern of the paresis, the dermatological findings, laboratory values, other organ manifestations, and the histomorphology of the muscle are considered. An overall score is derived, which is then converted into a percentage probability value and assigned to the probability categories. Those are a "high probability" (≥ 90%), "possible IIM/medium probability" (10–90%) and "low probability" (≤ 10%). For the purpose of comparability with the ENMC system, patients with a percentage probability below the cut-off level of ≥ 55% are also assigned and classified.

### Statistical analysis

We used descriptive statistics to describe demographic variables and categorical variables as statistical frequencies. Unless stated otherwise, the percentages refer to the entire cohort of 30 DM patients. To describe the metric variables, median, first and third quartile as well as maximum and minimum are given.

Due to the skewed distribution of almost all metric variables, non-parametric tests were applied regarding the comparison of the classification systems for group and pairwise comparisons. The Kruskal–Wallis test was used as the overall test for the comparisons between the four classification groups (ENMC: certain; probable; amyopathic; DM without dermatitis) and the EULAR/ACR score. In the case of a significant result in this test, the Mann–Whitney U test was used as a posthoc test for pairwise comparison. In order to be able to make a statement about their agreement, the Cohen's Kappa (κ) agreement measure was collected in relation to the criteria probabilities (ENMC: certain; probable / EULAR/ACR: high; medium).

Statistical analyses were performed using IBM SPSS Statistics 26 statistical software. *p* values < 0.05 were considered statistically significant.

## Results

### General findings

As part of the review of the diagnostics carried out and their findings, it was possible to describe statements on the diagnostic procedures used as well as the various clinical features of the DM patients. Table 1 shows an overview of those results.

**Table 1:** Clinical features of patients with dermatomyositis

Age (at diagnosis)	54.23 years	ANA positivity	12 (40%)
Sex (female: male)	2.3:1	DM-specific antibodies *(n* = *17)*	12 (70.5%)
Proximal muscle weakness	29 (96.7%)	Anti-Jo1 positivity *(n* = *28)*	0
		Ro52 *(n* = *27)*	10 (37.0%)
Raynaud	2 (6.7%)	Elevated CK *(n* = *24)*	16 (66.7%)
Dysphagia	6 (20.0%)		
Arthralgia/arthritis	11 (36.7%)	*Cutaneous involvement*	
ILD	5 (16.7%)	Heliotrope rash	27 (90.0%)
Cancer	8 (26.7%)	Gottron’s papules	6 (20.0%)
		Gottron’s sign	0
*Clinical diagnosis*			
Definite DM	14 (46.7%)	*Muscle biopsy (n* = *24)*	
Probable DM	12 (40.0%)	Endomysial inflammation	13 (54.2%)
Amyopathic DM	1 (3.3%)	Perimysial/perivascular infl	7 (29.2%)
DM sine dermatitis	3 (10.0%)	Perifascicular atrophy	16 (66.7%)
		Rimmed vacuoles	1 (4.1%)
*Criteria probability*			
High	16 (53.3%)		
Medium	13 (43.3%)		
Low	1 (3.3%)		

*ILD* interstitial lung disease, *DM* dermatomyositis, *ANA* antinuclear antibody, *CK* creatine kinase

### Patient classification according to ENMC (2003)

Among the 30 patients considered, 46.7% (n = 14) could be classified as "definite" and 40% (n = 12) as "probable dermatomyositis". Only one patient was classified as "amyopathic dermatomyositis" and three (10%) as "possible DM *sine dermatitis*".

### Patient classification according to EULAR/ACR (2017)

Regarding the entire patient cohort, including patients with and without muscle biopsy, the median value of the probability score is 9.1 with a range of 5.1–13.4 (Q1: 7.28 / Q3: 11.45). This corresponds to a "high" probability of diagnosis.

24 out of 30 DM patients (80%) had received a muscle biopsy. Regarding the total score of these patients, the median value is 9.95 with a range of 5.1–13.4 (Q1: 7.83 / Q3: 12.28). This corresponds to a percentage probability of about 88% reflecting a "high" criteria probability.

Six patients without a muscle biopsy taken reached a median value of 7.2 with a range of 6.0–8.0 (Q1: 6.53 / Q3: 7.48). This corresponds to a percentage probability of about 82% reflecting a "medium" criteria probability.

### Classification systems in comparison

As the results of the two approaches suggest, this study shows a variety of possible combinations between the respective classification statements. Those eight combinations are listed in Table 2 and shown in number and percentage ratio:

**Table 2:** Comparison of the results of the classification systems

Comb	ENMC (2003)	EULAR/ACR (2017)	Amount	%	κ
1	Definite DM	High probability	11/30	36,7	0.400
2	Definite DM	Possible IIM	3/30	10	–
3	Probable DM	High probability	4/30	13,3	–
4	Probable DM	Possible IIM	8/30	26,7	0.324
5	Possible DM sine dermatitis	High probability	1/30	0,03	–
6	Possible DM sine dermatitis	Possible IIM	1/30	0,03	–
7	Possible DM sine dermatitis	Low probability	1/30	0,03	–
8	Amyopathic DM	Possible IIM	1/30	0,03	–

*ENMC* European Neuromuscular Centre, *EULAR *European League Against Rheumatism,* ACR* American College of Rheumatology,* DM* dermatomyositis,* IIM i*diopathic inflammatory myopathies

Combinations 1 and 4, which are congruent in their statements, make up the majority of patient cases here (n = 19/30). As can be seen in the table, there is a heterogeneous range of combinations. The agreement between the classification probabilities of "definite/high" (κ = 0.400) and "possible/medium" (κ = 0.324) was fair.

**Fig. 3:**
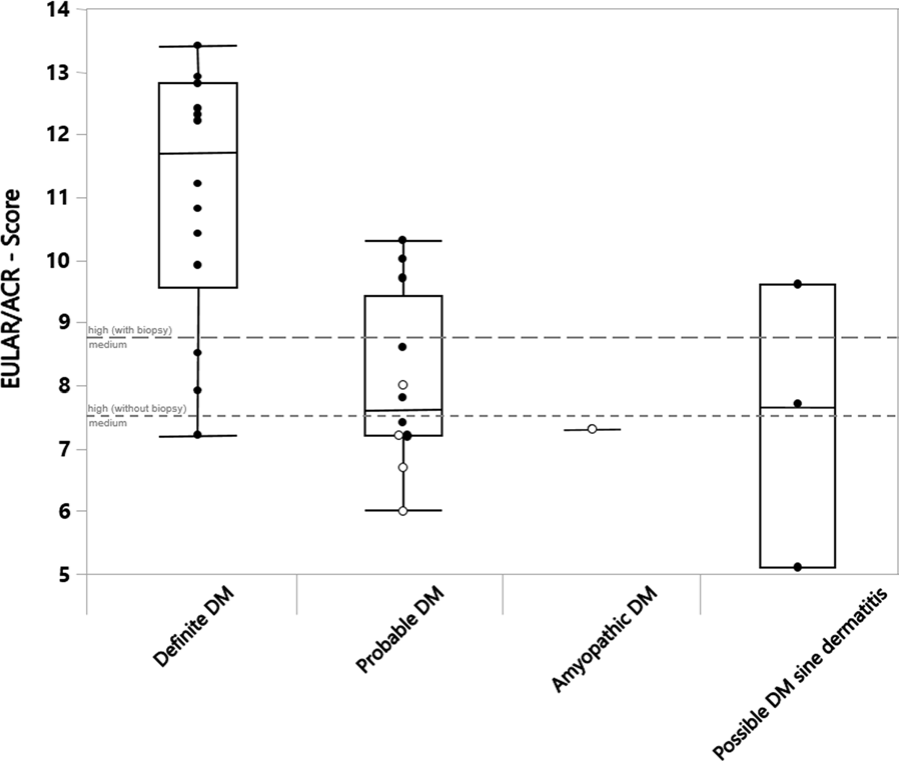
Boxplot diagram of the various DM-ENMC subtypes (2003) and their EULAR/ACR score calculated using the Kruskal–Wallis test. Cases with muscle biopsy are indicated by closed circles and cases without muscle biopsy by open circles

In Fig. 3 the individual ENMC subclasses can be compared with the EULAR/ACR score. For "definite DM", the median score is 11.7 with minimum and maximum scores of 7.2 and 13.4. "Probable DM" was characterized by a median score of 7.7 with minimum and maximum scores of 6.0 and 10.3. "Amyopathic DM" was only found in one patient; therefore, the EULAR/ACR score of 7.3 reflects a single value. The median score of 7.7 together with the minimum and maximum scores of 5.1 and 9.7 describes the relationship between "possible DM *sine dermatitis*" and the EULAR/ACR score.

Furthermore, there is a significant difference (p = 0.004) between the individual subclasses of the ENMC in relation to the EULAR/ACR scores. The "definite DM" has a significantly higher score value than the "probable DM" (p = 0.001). In relation to "possible DM *sine dermatitis*" a significantly higher EULAR/ACR score was identified (p = 0.032). No significant difference could be shown in the combinations of the other ENMC subclasses in relation to the score value.

## Discussion

The retrospective application of two different classification systems to patients with dermatomyositis enables for the first time a direct comparison of the approaches. While there are many different publications dealing specifically with the comparison of the classification systems with the original approach of Bohan and Peter [7, 22], a direct comparison of the currently established systems on a single tertiary center cohort is missing. The aim of the study was to evaluate the existing classification systems regarding their practicability, advantages and disadvantages as well as their basic concepts. The definition of a harmonized evaluation system with homogeneous case groups and clearly weighted diagnostic factors provides the basis for adequate medical patient care as well as clinical studies.

### Advantages and disadvantages of classification systems

Practicability plays an important role and is crucial for a reliable diagnosis. Here it is defined by the quality of applying the classification systems retrospectively and simultaneously applying them into daily clinical use. In this respect, the EULAR/ACR approach can be favoured. The easily recognizable and clear structure of the classification [17] paves the way for structured diagnostics for every practitioner. Depending on the clinical and dermatological findings and additional review of laboratory measurements, a decision can be made as to whether a muscle biopsy is necessary. Nevertheless, it is important to distinguish between diagnostic criteria and classification criteria and to remember that classification criteria in the strict sense are not intended for diagnosis. They are developed based on findings in patients with established and well-defined disease and, when used as diagnostic criteria, can mislead the clinician.

Because the classification according to the "historical approach" of Bohan & Peter was not applied in this study and considering the small size of the patient cohort, no conclusions regarding sensitivity and specificity can be drawn here. At this point we refer to previously published data on sensitivity and specificity related to the two systems used [9, 20, 23]. In addition, we conclude that the diagnostic procedure of histopathological examination has a clearly positive effect on sensitivity and specificity.

Strengths and weaknesses can also be described in terms of focus and the criteria considered. The use of a minimum of clinical and readily available laboratory parameters seems questionable. The reason for this can be found in the emergence of the classification criteria [24]: The criteria were data-driven and based on patient and comparator data from many medical centers worldwide. A disadvantage is the weak and even often lacking consideration of diagnostic procedures such as antibody diagnostic, MRI or EMG. It must be said that especially the procedures of MRI and EMG considerably facilitate diagnosis, allow staging of the disease and can help in the selection of a suitable biopsy site. For this reason, Luu et al. showed that the inclusion of these procedures improves the accuracy of the probability of IIM diagnoses using the EULAR/ACR classification system [25]. However, the homogeneous distribution of the score points of the respective diagnostic parameters allows a wide range of observation of the patient cases [20]. Dermatological findings are considered highly significant and are characteristic or pathognomonic for dermatomyositis, except in special cases. For this reason, it is necessary to perform a muscle biopsy if the skin findings are inconspicuous or absent [26].

The ENMC criteria (2003) focus on muscle biopsy as the gold standard of diagnosis, combined with a set of clinical criteria [9]. A reliable diagnosis without the histopathological findings of perifascicular atrophy is inconceivable. It can be stated that there is no equal significance between the results of the diagnostic procedures and that the muscle biopsy is prioritized. At this point, reference should be made to the more recent DM-specific ENMC classification approach from 2018 [17]. This system follows the idea that DM autoantibodies are specific for DM and can be associated with a characteristic clinical phenotype, prognosis and response to treatment [27, 28]. The possibilities of antibody diagnostics for classification of myositides were also discussed by Mariampillai et al. in 2018 [29]. They were able to show that myositis-specific antibodies are mainly important in the classification of myositis subtypes, whereas pathological data might be dispensable. They also refer to the EULAR approach and add the presence of four adjusted myositis entities to the classification system. Unfortunately, due to the frequent lack of antibody testing in the patients in our retrospective study, this was impossible. Data on applicability, sensitivity or specificity must be evaluated in the upcoming years.

### Decision on classification according to ENMC and EULAR/ACR

The patient cohort was small but strictly selected. Because of the strict pre-selection, the classification scheme could have been influenced towards statements of higher criteria probabilities. Furthermore, the proportion of patients with "probable DM" must also be critically reflected upon, as this includes patients without a muscle biopsy. The possibility of "definite DM" remains open for half of the patients in this probability level due to the lack of a muscle biopsy.

Regarding the EULAR/ACR approach, the comparison of the two models "with" and "without muscle biopsy" must be discussed. Our results allow the statement that a muscle biopsy significantly increases the probability of a reliable diagnosis. The assessment of the criteria probability used here is slightly modified compared to the approach according to EULAR/ACR. The cut-off level of 55% was not considered here because of the aim of comparison. One patient case would have been excluded (Case-Nr. 13).

As already described, it can be asserted that patients tend to be assigned to the same criteria probability. This depends on their findings and independent of the chosen classification system. Adjusted for chance, the agreement in the sense of the definition according to Landis and Koch from 1977 [30] can be described as "fair".

A deviating classification of the diagnostic probability could be explained by the different structure of the systems. While the statement of a "definite" diagnosis according to ENMC is mainly based on the typical muscle biopsy and the clinical-dermatological statements, this combination of findings according to EULAR/ACR is only sufficient for a medium probability of criteria. Conversely, the ENMC classification cannot make any statements in the sense of a definitive DM in the absence of a muscle biopsy, but the EULAR/ACR approach can. The classification types of DM without dermatitis and amyopathic DM according to ENMC (2003) can be compared poorly with the statements according to EULAR/ACR, due to a lack of a statement on probability. Nevertheless, our study shows a heterogeneous range of combinations.

A general difficulty was the assignment of the clinical findings to the scoring system. The very precise definition of the parameters in combination with partially rather general descriptions allowed the possibility of wrong classifications and deviations in the score. In particular, the need for detailed documented clinical information led to a substantial reduction in the number of available cases.

## Conclusion

The retrospective application of the ENMC (2003) and the EULAR/ACR (2017) classification to patients with dermatomyositis enables for the first time a direct comparison of the approaches. We did not show any inferiority of one of the two systems but a muscle biopsy significantly increases the probability of a reliable diagnosis. The question arises whether the need for routine histopathologic examination is still contemporary in clinical practice or whether other methods like antibody diagnostics could partly replace them. Similar to what has already been described by Zhang et al. (2019), the EULAR/ACR system has a high potential for being the classification system of the future [22]. However, it is important to find a consensus of the involved medical disciplines and to establish a structured procedure. It is currently up to the clinician to decide which approach to use based on experience and routine. It is therefore necessary to adapt to the available findings in order to be able to offer adequate care and therapy to the patient. A revision of the criteria is recommended in a few years' time, when more consistent serological information is available [17].

## Data Availability

The datasets used and/or analysed during the current study are available from the corresponding author on reasonable request.

## Abbreviations

ACR: American College of Rheumatology
DM: Dermatomyositis
EMG: Electromyography
ENMC: European Neuromuscular Centre
EULAR: European League against Rheumatism
IBM: Inclusion body myositis
IIM: Idiopathic inflammatory myopathies
IMNM: Immune-mediated necrotising myopathy
MAC: Membrane attack complex
MRI: Magnetic resonance imaging
PM: Polymyositis
